# Changes of lipoxin levels during pregnancy and the monthly-cycle, condition the normal course of pregnancy or pathology

**DOI:** 10.1007/s00011-020-01358-6

**Published:** 2020-06-02

**Authors:** Małgorzata Szczuko, Joanna Palma, Justyna Kikut, Natalia Komorniak, Maciej Ziętek

**Affiliations:** 1 Department of Human Nutrition and Metabolomics, Pomeranian Medical University, Szczecin, Poland; 2 Department of Perinatology, Obstetrics and Gynecology, Pomeranian Medical University, Szczecin, Poland

**Keywords:** Inflammation, Lipoxin, Menstruation, Ovulation, Pregnancy, Reproduction

## Abstract

**Objective and Design:**

The purpose of the review was to gather information on the role and possibilities of using lipoxin in the treatment of infertility and maintaining a normal pregnancy. Ovulation, menstruation, embryo implantation, and childbirth are reactions representing short-term inflammatory events involving lipoxin activities. Lipoxin A4 (LXA4) is an arachidonic acid metabolite, and in cooperation with its positional isomer lipoxin B4 (LXB4), it is a major lipoxin in mammals. Biosynthesis process occurs in two stages: in the first step, the donor cell releases the eicosanoid intermediate; secondarily, the acceptor cell gets and converts the intermediate product into LXA4 (leukocyte/platelet interaction).

**Results:**

Generating lipoxin synthesis may also be triggered by salicylic acid, which acetylates cyclooxygenase-2. Lipoxin A4 and its analogues are considered as specialized pro-resolving mediators. LXA4 is an important component for a proper menstrual cycle, embryo implantation, pregnancy, and delivery. Its level in the luteal phase is high, while in the follicular phase, it decreases, which coincides with an increase in estradiol concentration with which it competes for the receptor. LXA4 inhibits the progression of endometriosis. However, during the peri-implantation period, before pregnancy is confirmed clinically, high levels of LXA4 can contribute to early pregnancy loss and may cause miscarriage. After implantation, insufficient LXA4 levels contribute to incorrect maternal vessel remodeling; decreased, shallow trophoblastic invasion; and the immuno-energetic abnormality of the placenta, which negatively affects fetal growth and the maintenance of pregnancy. Moreover, the level of LXA4 increases in the final stages of pregnancy, allowing vessel remodeling and placental separation.

**Methods:**

The review evaluates the literature published in the PubMed and Embase database up to 31 December 2019. The passwords were checked on terms: lipoxin and pregnancy with combined endometriosis, menstrual cycle, implantation, pre-eclampsia, fetal growth restriction, and preterm labor.

**Conclusions:**

Although no human studies have been performed so far, the cell and animal model study results suggest that LXA4 will be used in obstetrics and gynecology soon.

## Introduction

Data on inflammation in diseases is intensively sought at the same time, this process has not been studied in physiological conditions where it is an indispensable factor in the proper course of the menstrual cycle and implantation and delivery. That is why we have attempted to collect information and systematize it in the course of a correct and pathological reproductive cycle. Our review focuses on the role of AA-derived lipoxins whose homologues as separating mediators are used in the treatment of inflammatory diseases.

Inflammation is the body’s protective response against injury and infection and, when poorly controlled, it may develop into chronic inflammation with probable further tissue damage. The resolution of inflammation is crucial for tissue to return to homeostasis and has become a new space for research into the inflammation process. Inflammation may be controlled by local endogenous anti-inflammatory mediator production, including cortisol, interleukin 10 (IL-10), A1 protein annexin (ANEX-1) and lipid mediators such as resolvins, protectins and lipoxins (LX) [1–3]. Transient inflammation is a necessary reaction during the tissue remodeling and repair process, and in the case of uncontrolled evolution, it becomes a pathological phenomenon. Ovulation, menstruation, embryo implantation and childbirth are reactions representing short-term inflammatory events involving lipoxin activities [4]. The purpose of the review was to gather information on the role and possibilities of using lipoxin in the treatment of infertility and maintaining a normal pregnancy.

Lipoxin A4 (LXA4) is an arachidonic acid metabolite (C20H32O2) and, in cooperation with its positional isomer lipoxin B4 (LXB4), is a major lipoxin in mammals. Generating lipoxin synthesis may be also triggered by aspirin or salicylic acid, which acetylates (aspirin acetylates a serine) cyclooxygenase-2 (COX-2) [5]. Aspirin-triggered lipoxins (ALT) such as 15-epi-LXA4 or 15-epi-LXB4 are synthesized in this way [6]. The lipoxin 15-epi-LXA4 has a common ability to separate and angiogenesis with LXA4; however, its biochemical effect lasts longer [7].

Lipoxins as endogenous eicosanoids play an active role in the regulation of inflammation [8]. LXA4 biosynthesis is recognized as an intercellular process through interactions between different cells. The process occurs in two stages: in the first step, the donor cell releases the eicosanoid intermediate; secondarily, the acceptor cell gets and converts the intermediate product into LXA4 [9]. As many as three iron-containing enzymes called lipoxygenases—5-LOX, 12-LOX and 15-LOX—are involved in LXA4 biosynthesis. The leukocyte/platelet interaction with the participation of two key enzymes 5-LOX and 12-LOX is one of the most important synthesis pathways. To catalyze arachidonic acid into leukotriene A4, leukocytes use 5-LOX, where leukotriene A4 is further converted by 12-LOX into LXA4 in platelets. 5-LOX is also found to be active in neutrophils. In contrast, the lipoxygenation of arachidonic acid by 15-LOX takes place in epithelial cells or monocytes.

Lipoxin A4 can act and influence many types of cells, including blood cells, neurons and stromal cells [10]. LXA4 has been shown to block the production of IL-12 in dendritic cells by increasing the suppressor of cytokine-2 signaling expression [11]. In addition, it stimulates the phagocytosis of apoptotic cells by reprogramming macrophages from types M1 to M2 [12].

Lipoxin A4 and its analogues, such as BML-111, are considered as specialized pro-resolving mediators (SPMs) that “inhibit” inflammation signals. It is the first mediator to have dual anti-inflammatory and pro-resolution activities. Lipoxin A4 exhibits the selective regulation of the leukocyte response by activating the specific ALXR receptor (also called FPR) in neutrophils. The interaction between LXA4 and ALXR blocks the migration and neutrophil penetration of inflammation sites. LXA4 strongly inhibits leukocyte inflow into inflammatory sites by binding the high-affinity G-protein coupled receptor and stimulates the phagocytosis of apoptotic cells by macrophages [13]. In contrast, in monocytes, the LXA4–ALXR interaction stimulates monocyte recruitment and the phagocytosis of apoptotic neutrophils. Additionally, it has been found that ALXR (also called FPR) plays a significant role as a pleiotropic regulator in macrophage polarization [14]. In humans, three FPR protein paralogs have been identified (FPR1, FPR2 and FPR3). Their activation by specific agonists leads to transient calcium streams, signal-regulated extracellular kinase (ERK) and chemotaxis [15]. Of these, FPR2 is unique in that it uses both lipid and protein ligands. The majority of FPR2 agonists are peptides, except for eicosanoid, LXA4 and synthetic ligands, which, by activating FPR2, improve basic cellular functions, including proliferation, differentiation, invasion, angiogenesis, and regulating the inflammatory response [16].

**Fig. 1:**
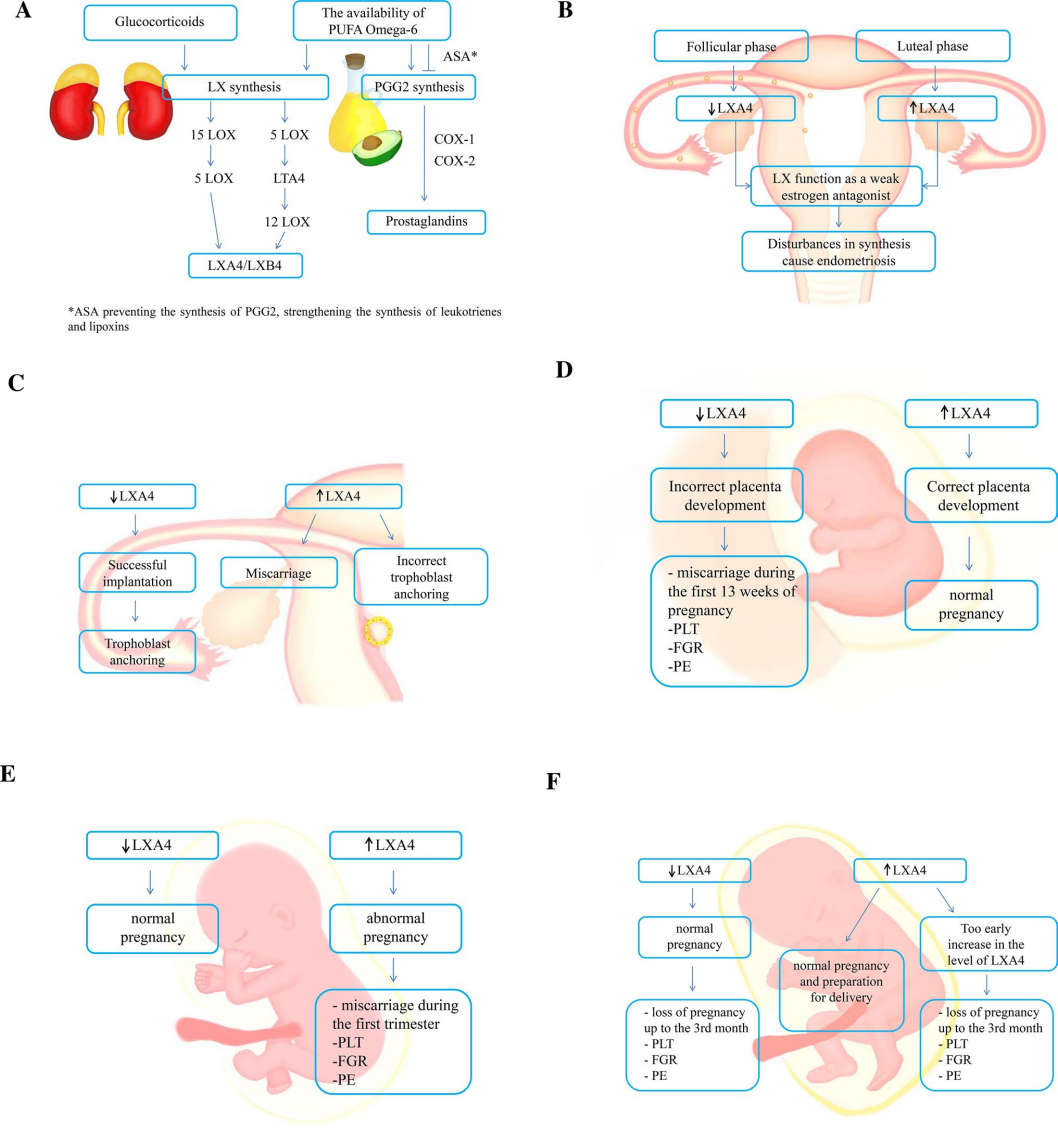
The role of lipoxins in the menstrual cycle and pregnancy. **a** Lipoxin synthesis, **b** Menstrual cycle, **c** Embryo implantation/trophoblast anchoring, **d** 1st trimester - placenta development, **e** 2nd trimester - fetus development, **f** 3rd trimester - preparation for delivery

It has also been shown that glucocorticoids (GC) may promote the initiation of placental inflammation and delay recovery by disrupting LXA4 biosynthesis [17]. Exposure to GC may be associated with obstetric complications, including preterm delivery, intrauterine growth restriction (IUGR), and pre-eclampsia (PE) [18]. Abnormal GC metabolism is also responsible for spontaneous miscarriages [19]. GC activity is mediated by the calcium-dependent phospholipid binding protein, Annexin A1 (ANXA1) [20]. Nevertheless, the potential applications for lipoxin A4 in the area of reproductive biology remain unexplored and require further research (Fig. 1a).

## Lipoxin A4 in the menstrual cycle

In menstrual cycle species, such as humans and most of primates, the cyclical sloughing of degraded endometrial tissue of the upper two-thirds of the endometrium requires rapid healing and regeneration while maintaining defense mechanisms and minimizing inflammatory reactions [21]. Regular LXA4 level changes have been found during the menstrual cycle in women, indicating the occurrence of menstruation-related inflammation reaction [22]. In the endometrium zone, inflammatory leukocytes are thought to play an important role in the tissue necrosis process and endometrial remodeling, which is necessary for normal menstruation. Chronic uncontrolled inflammatory reactions in the endometrium are found to be associated with several pathologies, including severe menstrual blood loss [23]. In investigation by MacDonald et al., a potential role and expression of A4 lipoxin and FPR2/ALXR receptors related with inflammatory process regulation was elucidated, while the cyclic endometrial remodeling in humans during menstrual cycle was evaluated. LXA4 levels rise in the end of the luteal/secretory phase of menstrual cycle, and then decrease during menstruation in the proliferative/follicular phase [24]. This coincides with an increase in estradiol (E2) levels and LXA4 competition for estrogen receptors (ERs) [25]. Such a cycle ensures the reproductive success of our species, where lipoxin is triggered by aspirin, which regulates inflammation and the sensation of pain, which plays an important and special role (Fig. 1b) [26].

## Lipoxin role in endometriosis

The endometrium is a dynamic activity tissue that undergoes physiological changes during the menstrual cycle as a result of ovaries’ steroid hormone interaction. Stimulated by estrogens, a rapid thickening of the functional layer accompanied by angiogenesis occurs. Blood vessels and nerves use common genetic signals and pathways for growing and function regulation [27]. LX, as a strong angiogenesis modulator, is believed to be involved in endometrial neuroregulation [6]. LXA4 inhibits the progression of endometriosis and, as an endogenous SPMs and immunomodulating mediator, is a potentially significant factor in endometriosis development [28, 29]. A hypothesis assumes that LXA4 acts as an estrogen receptor agonist in endometrial epithelial cells. Antagonizing some of the estrogen-mediated effects in a manner similar to low-activity estrogen, with which it has structural similarity, it lowers the growth of the functional layer of the endometrium [4]. It has been found that both endometrium coming from experimental endometriosis induced in rats and ectopic endometriosis from human tissue showed a higher expression of the LXA4 receptor when compared to normal tissues. In contrast, the expression of leukotriene B4 receptors (BLT1 and BLT2) did not change in endometriosis. These observations suggest a possible role of LXA4 and its receptor in the physiological cycle of oestrus and pathological inflammation, as well as estrogen-dependent conditions such as endometriosis [29]. Moreover, aspirin also triggers the generation of epimeric forms such as 15-epi-LXA4 and 15-LOX type 2, which show a substrate preference for arachidonic acid metabolized to 15S-hydroperoxyeicosatetraenoic acid (15S-HETE) in the human endometrium [25].

## Role of lipoxin A4 in pregnancy regulation

Pregnancy is increasingly recognized as an inflammatory process influenced by pro-inflammatory, SPMs and anti-inflammatory modulators in various gestation phases [13]. Primarily, pro-inflammatory cytokines contribute to blastocyst implantation [30]. The pro-inflammatory response is therefore favorable at the beginning of pregnancy. Moreover, VEGF receptor 1 (Flt-1) disables proteins that cause blood vessel growth and seems to play a detrimental role during embryogenesis. PlGF is a member of the VEGF family and stimulating angiogenesis: PlGF interaction with its receptor can be inhibited by sFlt-1, resulting in endothelial dysfunction. PlGF playing a key role in the formation of the placenta. In normal pregnancy, the serum concentration of PlGF increases from 8–12 weeks and reaches a peak at 29–32 weeks, then decrease to 33–40 weeks gestation [31]. However, the further course of pregnancy is characterized by an anti-inflammatory condition that persists up to mid-pregnancy, from the time it evolves back again to inflammation status in the last trimester of gestation and before labor. Studies have shown that low LXA4 levels during blastocyst implantation and labor, as well as LXA4 high levels in the mid-pregnancy, are beneficial for the proper course of blastocyst implantation, pregnancy maintenance and labor. In addition, Xu et al. provided evidence that spontaneous miscarriages are associated with the stress/glucocorticoid/lipoxin A4 axis [19].

A controlled inflammatory environment is beneficial for the start and maintenance of pregnancy, while an uncontrolled inflammatory response may be detrimental for its further course [10]. The inflammatory response is usually self-limiting and plays a key role in maintaining tissue homeostasis [32]. However, it can develop into chronic stage once the programming process fails. To avoid the catastrophic consequences of the uncontrolled inflammatory response, comprehensive mechanisms such as the production of the anti-inflammatory cytokines IL-10 and TGF-b and metabolic enzyme indoleamine 2, 3-dioxygenase or arginase 1 have been developed [33]. Endogenous LXA4 is therefore a key factor in maintaining a normal pregnancy by regulating inflammation-related factors, mast and others cells. As a major inflammatory factor, mast cells release various inflammatory mediators, including histamine, proteases and cytokines such as IL-6, IFN-γ. LXA4 may regulate mast cell migration, inhibit proinflammatory factors (including iNOS, CCL2, IFN-γ and IL-6) and at the same time increase IL-10 expression in the placenta [34]. It has been also found that the level of decrease of the key enzyme inactivating glucocorticoid activity (GK), 11β-hydroxysteroid dehydrogenase (HSD) type 2, inhibits LXA4 biosynthesis, leading to early pregnancy loss. Therefore, glucocorticoids may be identified as a molecular basis underlying the decrease in LXA4 levels during miscarriage. Their use in the chorionic villus or placenta is significantly increased while systemic cortisol levels do not fluctuate [19]. Moreover, a type of corticosteroid, dexamethasone, influences the enhancement and activation of the mitogen-activated protein kinase (MAPK) and nuclear factor kappa-light-chain-enhancer of activated B cells (NF-κB) pathways in lipopolysaccharide (LPS)-stimulated (low-dose endotoxin) rat placenta [17]. When taking into consideration the classic evolutionary perspective, miscarriage should not be defined as a sickness, but as an effective protective mechanism, ensuring the survival of the strongest species individuals.

## Embryo implantation

Embryo implantation is achieved due to the delicate balance between pro-, SPMs and anti-inflammatory mediator production in order to apply the required tissue function and homeostasis. It is well known that abnormal trophoblast invasion and the pathologic spiral arteries remodeling process are strongly related to placental ischemia and hypoxia, leading to further medical complications.

LXA4, as an SPMs, interferes with the implantation process; therefore, its activity is disadvantageous for the course of pregnancy at this stage. Furthermore, LXA4 has a time- and dose-dependent effect on embryo implantation, while the blocking embryo implantation is made possible by controlling estrogen receptor α (ERα) activity [35]. A study conducted by Xu et al. also proved that LXA4 interferes with implantation by mesenchymal epithelium (EMT) secretory activity inhibition. Affecting many important factors, including matrix metalloproteinases (MMP9), β-catenin, vimentin, Akt, Gsk-3β and NF-κB, the LXA4 reduces integrin linked kinase (ILK) regulation through an increase in FPR2 receptor concentration [35]. Moreover, LX may regulate the expression of γ-aminobutyric acid (GABAAπ) endometrial receptor throughout homeobox A10 (HOXA10), and finally may control the secretion of leukemia inhibitory factors (LIF) and cardiotrophin (CT) by the endometrial gland during implantation [36, 37]. As is known, LIF acts on human trophoblast by changing its differentiation towards anchoring phenotype throughout the increase in fibronectin synthesis [38]. However, blocking the LXA4 signaling pathway in later periods may cause miscarriage. Based on the above findings, the LXA4, which acts on embryo implantation by inhibition, could be used as a new contraceptive agent (Fig. 1c).

## The first trimester of pregnancy

An increase of LXA4 concentrations during early pregnancy is probably caused by human chorionic gonadotropin secretion (hCG), because the stimulation of human decidual tissue hCG release is related with an increase of LXA4 [38]. In this way, the release of LXA4 in the human endometrial decidual layer after embryo implantation is promoted, which has been also described by other authors [24]. Although the usefulness of ATL (aspirin-dependent lipoxin) in the field of reproduction has not been extensively studied, some reports suggest the possibility of its use in this context. An increase of receptor expression (ALXR) has been also observed in the decidual layer in the first trimester of gestation [39]. According to other studies, early administration of low-dose aspirin in pregnancy (before the 16th week of gestation) provides an 89% reduction of pre-eclampsia (PE) incidents occurring before the 37th week of gestation. On the other hand, the PE risk remains at a constant level when aspirin is given after 16 weeks of pregnancy. These results suggest that ASA has a beneficial effect on the pregnancy course until chorion formation is completed (before the 16th week of gestation), and then further ASA administration does not change the course of late pregnancy [40].

Early human pregnancy develops in a low oxygen content environment, which limits reactive oxygen species (ROS) production and is necessary for proper fetal growing. However, the oxygen concentration increases rapidly towards the end of the first trimester, when the fetoplacental circulation is established, causing an imbalance and increase of placental oxidative stress [41]. Then, mRNA of antioxidant enzymes and their activity superoxide dismutase (SOD) and peroxidase glutathione (GPx) increase in order to remove the emerging ROS [42]. If ROS generation exceeds the antioxidant defense capacity, oxidative stress occurs, which plays a key role in placenta-related diseases development, such as fetal growth restriction (FGR) and pre-eclampsia (PE) [43]. Therefore, moderate apoptosis is necessary for the normal development of the placenta, but excessively increased apoptosis in the placenta is usually associated with pregnancy abnormalities and complications (Fig. 1d).

## The second trimester of pregnancy

The lipoxin A4 level in pre-eclampsia is found to be much lower than in normal pregnancy; therefore, insufficient production of lipoxin A4 after embryo implantation may lead to immunomicro-energetic placental abnormalities. In addition, lipoxin A4 suppresses an increased production of proinflammatory cytokines and alleviates the symptoms of inflammation, contributing to a normal pregnancy course. Because both estrogen and progesterone are key hormones in maintaining pregnancy, LXA4 plays a significant role in regulating pregnancy by competing for their common receptor (Fig. 1e).

## The third trimester of pregnancy, labor

The effect of LXA4 on LPS-induced oxidative stress in human umbilical vein endothelial cells (HUVEC) has been studied by Liu et al. [44]. LXA (4) has been found to increase the level of enzymes such as nicotinamide adenine dinucleotide quinone oxidoreductase (NQO1) and heme oxidase 1 (HO-1) to protect HUVEC against oxidative stress induced by LPS. In addition, immunofluorescence assay results showed that, in the control group, the nuclear factor erythroid -2- related factor 2 (Nrf2) protein, which plays a key role in cell protection against the harmful effects of oxidative stress, was expressed in the cytosol but not in nucleus. In the LPS analyzed group, the expression of Nrf2 and quinone oxidoreductase (NQO1) proteins strongly decreased in the nucleus and cytosol, while in the LPS and LXA4 analyzed groups, the expression was significantly higher compared to the LPS group [44]. Assuming that reactive oxygen species (RFT) cause the dissociation of Nrf2 from an inactive complex with Keap1, resulting in Nrf2 translocation to the nucleus and the induction of multiple cytoprotective gene expression involved in RFT degradation, the deactivation of electrophilic metabolites, the detoxification of xenobiotics, and the stabilization of the cell oxidoreduction potential, LXA4 plays a significant role in regulating the inflammatory response [45]. Human placental expression of the lipoxin A4 FPR2/ALX receptor has been also demonstrated since the third trimester of gestation; however, the increased LXA4 level has been observed to be lower when compared to pro-inflammatory cytokines, such as IL-1β and TNF-α [46]. Inflammatory processes are crucial for several pregnancy events, while the dysregulation and imbalance of inflammation is a typical feature in pregnancy complications such as pre-eclampsia (PE), fetal growth restriction (FGR) and preterm labor (PL) (Fig. 1f).

## Pre-eclampsia

Pre-eclampsia (PE) is a potentially dangerous pregnancy complication, affecting 2–7% of all gestations, although its incidence in developing countries may be higher [32, 47]. The pre-eclampsia pathogenesis is very complex, and includes genetic, immunological and environmental factor interactions. Pre-eclampsia is strongly related with placental malfunction, which develops between the 8th and 16th week of gestation. As a consequence of inadequate trophoblast invasion and insufficient uterine spiral artery remodeling, a superficial placental attachment leads to dysfunction, causing hypoxia and increased oxidative stress [48], consequently leading to a maternal systemic inflammatory response characterized by increased inflammatory mediator expression [49]. PE occurs in the second or third trimester of gestation and is characterized by maternal systemic symptoms such as hypertension, proteinuria, endothelial dysfunction (due to proinflammatory cytokines) and placental malfunction resulting in intrauterine fetal growth restriction. Severe complications of pre-eclampsia include cerebral hemorrhage, renal failure and HELLP syndrome (hemolysis, elevated liver enzymes and low platelet levels) [50]. It is also associated with a significant incidence worldwide of maternal and fetal mortality and morbidity in the perinatal period, particularly with a higher risk for heart diseases appearance in later life. Therefore, any therapy that reduces the risk of PE appears to be justified.

Various molecular mechanisms associated with abnormal placental development are observed, including the renin-angiotensin system, 1,25-dihydroxyvitamin d and lipoxin A4, while vacuolar ATPase (V-ATPase) is closely related to all three mentioned above [51]. It seems that any dysregulation of molecular events between V-ATPase and the renin-angiotensin system, 1,25-dihydroxyvitamin d and lipoxin A4 will have a negative effect on placental homeostasis.

Increased oxidative stress, reduced antioxidant capacity and increased levels of cellular calcium are the initial factors that affect endothelial cell permeability (EC) and are a key event in the pathogenesis of preeclampsia [52]. The counteracting effect of LXA4 in human umbilical vein endothelial cells (HUVEC) is based on the following:

- Blocking the production of reactive oxidative forms by promoting the expression of factor 2 protein (Nrf2). Nrf2 translocate into the nucleus and sequentially upregulates several further phase II enzymes, such as nicotinamide adenine dinucleotide (NAD) quinone oxidoreductase (NQO1) and heme oxygenase 1 (HO-1), which have appeared as important mediators of antioxidant and cytoprotective effects [53].
- The regulation of the correct cadherin and catenin expression, which reduce excessive permeability for endothelial cell lipopolysaccharide (EC).
- The inhibition of elevated cellular calcium levels and regulation of a family of transient potential C1 receptor proteins—an important calcium channel in the EC.

Pang et al. also found that LXA4 prevents LPS-induced EC hyperpermeability in human umbilical vein endothelial cells (HUVEC), involving pathways sensitive to Nrf2 as well as Ca2 + [54]. LXA4 inhibits LPS-induced hyperpermeability in HUVEC mainly throughout the ALXR receptor, although other receptors, such as cysteinyl-leukotriene receptors, and growth factor receptors may also mediate this reaction [55, 56]. Interestingly, Huang et al. proved that LXA4 levels in women with mild pre-eclampsia were significantly higher than in the control group [57]. However, no significant statistical differences were found between normal gestation and severe pre-eclampsia. In addition, LXA4 receptor mRNA expression was significantly lower in women with pre-eclampsia than in the control group (*p* < 0.01), and NF-kappa B p65 mRNA expression was significantly higher in pre-eclampsia (*p* < 0.01). It is possible that an increase of LXA4 level in mild pre-eclampsia was present, while in severe pre-eclampsia, the greater use of LXA4 in the inflammation processes resulted in the exhaustion of the pool and lowering of its level to comparable values to the control group. Other authors have tried to explain this relationship by the variety of diet used and/or hormones [32].

Soluble vascular endothelial growth factor receptor (VEGFR)-1 levels, also known as sFlt1 (soluble fms-like tyrosine kinase 1), have been found to be elevated in pre-eclampsia. Therefore, pre-eclampsia may develop when an imbalance between pro and anti-angiogenic factors occurs. VEGF, which is an angiogenic factor, is necessary for the transport of polyunsaturated fatty acids (PUFAs) to endothelial cells. Hence, reduced VEGF levels reduce the availability of PUFAs for EC cells [50]. Moreover, it was noted that MicroRNA has potential role in the development of PE, with both increased and decreased expression in placenta [58]. Were found overexpressed in PE miR-16, miR-26b, miR-29b, miR-335, miR-222, miR-181a and miR-195,which are known to modulate the expression of VEGF and other angiogenic factors, placentas and determined a down-regulation of that target genes [59]. The last data reported that miR181 acts as modulator of T-cell sensitivity and selection and concurs to the tolerance induction at the maternal–fetal interface, which is disturbed during PE [60]. It is noteworthy that PUFAs form precursors of strong SPMs and anti-inflammatory molecules, including lipoxin, resolvin, protectin and maresin. They all have a vasodilating effect, prevent platelet aggregation, inhibit the production of pro-inflammatory interleukin-6 (IL-6) and tumor necrosis factor-α (TNF-α) and increase the synthesis of endothelial nitric oxide (eNO) [50]. In other studies, in the severe PE group lipoxin A4 inhibited increased tumor necrosis factor alpha (TNF-α) and interleukin-1β (IL-1β) production in monocytes in a dose-dependent manner by inhibiting extracellular calcium influx [61]. LXA4 demonstrates such properties in other cells as well [62]. Plasma IL-1β, CRP, IL-6, TNF-alpha, IL-15, IL-16 and IL-10 levels have been also reported to be increased in PE [63–65].

It is suggested that the increase in LXA4 and AnxA1 in the circulation of women with PE may act as a compensatory mechanism in the treatment of inflammation [32, 46, 66]. In contrast, LXA4 deficiency may cause pre-eclampsia, which can be attributed to a decrease in inflammatory response, oxidative stress, and regulation of 11-β-dehydrogenase isozyme 2 (11β-HSD2) corticosteroid [13]. Lipoxin A4 is therefore a promising molecule that can be used for the treatment of many inflammatory diseases, due to its strong protective effect, as demonstrated in various experimental animal disease models [67, 68]. Cadavid et al. indicate also the possibility of ATL using as a complementary therapy in women with PE or gestation related antiphospholipid syndrome (APS) [69]. In addition, they propose, regardless of the use of conventional anti-asthmatic and anti-allergic drugs in pregnancy, such as inhaled corticosteroids, long- and short-acting β-agonists, leukotriene modifiers, cromolyns and theophylline, to include anti-inflammatory, pro-resolution mediators and SPMs to the treatment protocol, such as lipoxins, resolvin and protectins [70]. It has been shown that the synthetic lipoxin analog BML-111 has a potential therapeutic effect in pregnant rats with induced PE, which has been linked with the inhibition of inflammatory processes in the placenta [71].

In conclusion, PE at the molecular level is induced by circulatory system factors that are derived from an abnormal placenta [51] and antiangiogenic sFlt1 which binds VEGF and placental growth factor (PlGF) [72]. These defects impair normal fetal and maternal vascular system development and cause placental ischemia and hypoxia, which contributes to the dangerous pathogenesis of PE.

Thus, pre-eclampsia is a vascular disorder involving pathogenic features of increased inflammation, oxidative stress, and endothelial dysfunction leading to medical complications [73]. A systematic review of 59 clinical trials (37,560 women) investigating antiplatelet therapy to prevent pre-eclampsia showed that low-dose aspirin reduced the risk of pre-eclampsia by approximately 17% [74]. A similar medical effect should therefore be expected with use of LXA4 analogues; however, such studies have not been performed in humans so far.

## Antiphospholipid syndrome (APS)

Antiphospholipid syndrome (APS) is an autoimmune disorder characterized by the presence of antiphospholipid antibodies (aPL) and is associated with clinical signs of thrombosis, blood disorders, including thrombocytopenia and hemolytic anemia, and/or pregnancy and fetal complications [75]. Women with high levels of aPL antibodies are at high risk of fetal loss in early and late pregnancy, intrauterine growth restriction (IUGR), placental insufficiency and pre-eclampsia [76].

aPL is a heterogeneous group of autoantibodies directed against a variety of negatively charged phospholipids located on cell membranes [77]. They can recognize phospholipid binding proteins such as annexin V, C and S proteins, prothrombin and Beta 2 glycoprotein I (β 2 GPI) [78]. However, it has been recently suggested that APL reacting with β 2GPI are the most pathological autoantibodies in gestational APS [79]. In addition, trophoblast cells can potentially bind to β 2 GPI from the circulatory system, synthesize their own β 2 GPI and express them in the plasma membrane as well, turning the placenta into a target for anti-β 2 GPI antibodies [80]. However, it is discussed that pregnancy-related aPL adverse effects are mainly caused by inflammatory processes and placental insufficiency at the mother’s site connection, rather than thrombotic processes [80].

aPL directed against β 2 GPI impair normal trophoblast function and thus causes adverse pregnancy outcomes. The extravillous trophoblasts normally invade the uterus and remodel the spiral arteries to enable increased blood flow. This transformation ensures the adequate angiogenesis of the placenta. It was found that, in PE, IUGR and APS complicated pregnancies, and defective decidual endovascular trophoblast invasion with pathological transformation of the spiral artery is a major cause of adverse pregnancy outcomes [81].

Currently, pregnant women with APS are treated with low-molecular-weight heparin (LMWH) as a monotherapy or in combination with a low-dose aspirin [82].

In endothelial cells, ATL can reduce reactive oxygen species production, differentially regulate neutrophil and monocyte chemotaxis, and inhibit NF-κB activity and pro-inflammatory cytokine production in immune cells as well [83]. The down-regulation of TNF-α and IL-8 by LXA4 may occur through the NF-κB pathway [84]. One of the limitations of endogenous lipoxins is their rapid metabolism and inactivation. However, stable ATL analogues have longer biological activity and can therefore be a better therapeutic option [10]. Systolic blood pressure, 24 h urinary albumin excretion, serum TNF-α and IL-8 levels, and morphological damage to the placenta and kidneys caused by LPS have been shown to be effectively alleviated by BML-111 (a synthetic LXA4 analogue also called heptanoate 5 (S), 6 (R) -7-trihydroxymethyl) [85].

## Fetal growth restriction (FGR)

Severe fetal growth restriction (FGR) is defined as a sonographic estimation of fetal weight below the fifth percentile for a given gestational age. Evidence of additional pathologies, including asymmetric fetal growth and reduced amniotic fluid index, allows obstetricians to differentiate FGR and fetuses small for gestational age (SGA) who are healthy [86]. Growth-restricted fetuses are at increased risk of developing metabolic disorders such as obesity, type 2 diabetes and cardiovascular diseases in their later life [87]. There is evidence that FGR is associated with oxidative stress [88]. Therefore, it was found that synthetic analog LXA4 (BML-111), administered as an antioxidant therapy, promotes the nuclear translocation of nuclear factor 2 associated with erythroid 2 (Nrf2), upregulates the expression of antioxidant enzyme genes, superoxide dismutase (SOD) and peroxidase glutathione (GPx) and, consequently, inhibits the apoptosis of trophoblast cells [89].

The hypothesis that LXA4 may modulate the inflammatory response in the early pregnancy implantation process and affect intrauterine fetal growth was proposed by Lipa et al. [90]. The investigators examined 189 patients and found that LXA4 concentration values may potentially be used in the first trimester of pregnancy as an early marker for FGR prognosis. The selective miRNA expression appears to be another FGR marker acting via an epigenetic mechanism [58]. According to the presented data, LXA4 concentrations were reduced in both hypo- and hypertrophic fetal groups [90]. Furthermore, increased trophoblast secretion activity and apoptosis associated with FGR pregnancies have been observed [91]. Lappas et al. also suggest a new trophoblast apoptosis mechanism in FGR-complicated pregnancies [92]. It is likely that the affinity of FPRK2 for lipoxin A4 and/or annexin A1 influences the activation of serine/threonine kinase, specifically the PI3K-Akt/PKB (protein kinase B) or MEK/ERK (mitogen-activated protein kinase) signaling pathways, as is the case for cancer cells characterized by increased proliferation, reduced apoptosis, and increased survival [16]. It was demonstrated that FPR2/Fpr2 expression in the placenta was significantly reduced in FGR pregnancies when compared to controls.

Intracellular stress signals are mediated by an imbalance of Bcl-2 (antiapoptotic) and Bax (proapoptotic) family proteins, which causes changes in mitochondrial membrane permeability. When the Bax protein is in excess, signaling pathways triggering apoptotic cell death are activated, but in Bcl-2 domination conditions, the signal is inhibited, and the cells survive [93]. These reactions were used by Lappas et al., who also demonstrated an increase of differentiation marker levels, chorionic gonadotropin beta (HCGB) and syncytin-2; cytokines, interleukin (IL) -6, CXCL8; and apoptotic markers such as TP53, caspase 8 and BAX in the FGR group [94]. At the same time, the expression of CXCL12 chemokines and its CXCR4, CXCR7, CXCL16 and CXCR6 receptors significantly decreased. Treatment with HUVEC siFPR2 significantly reduced proliferation, endothelial formation and significantly increased HUVEC-umbilical vein endothelium permeability as well [94].

Therefore, reduced FPR2 expression in FGR may have a medical significance, contributing to the premature depletion of the VCT proliferating pools, favoring their premature differentiation into ST, and leading to increased trophoblast secretion activity and apoptosis in FGR [95, 96].

## Preterm labor (PTL)

Preterm labor, defined as a birth occurring before the 37th week of gestation, remains an important obstetrical problem. PTL is estimated to affect around 5–18% of pregnancies worldwide, consequently leading to the preterm births of about 15 million of babies each year [97]. In addition, preterm birth is associated with an increased risk of several short-term diseases and long-term dysfunctions, including cerebral palsy, bronchopulmonary dysplasia (BPD), retinopathy of prematurity, and difficulties in later school education [98]. Although the direct cause of PTL occurrence is often unclear, in many cases, there is a relationship between PTL and the presence of latent or symptomatic intrauterine infection [99], in which premature inflammatory pathways activation is probably the most important reaction.

However, one of the first studies analyzing pro-inflammatory causes of preterm labor and changes in gene expression responsible for the synthesis of mediators caused by bacteria or ovarian resection showed that preterm labor caused by bacterial infection has significantly increased the expression of genes involved in prostaglandin synthesis. Moreover, preterm labor caused by ovariectomy increased the expression of genes involved in the synthesis of lipoxins, leukotriene and hydroxyeicosatetraenoic acid [100]. For the first time, the potential role of ALXA4 and its receptor (FPR2/ALX) in physiological and pathological inflammatory reaction during labor was shown by Maldonado-Pérez et al. [101]. The investigators demonstrated that LXA4 significantly reduced LPS-induced but not the basal expression of pro-inflammatory cytokines IL-6 and IL-8 in human myometrium during pregnancy. They also suggested that the production or administration of exogenous lipoxins as therapeutic agents could be successful strategies in the prevention of the inappropriate onset of inflammatory pathways resulting in preterm births [102].

Although the mechanism by which 15-epi-lipoxin A4 reduces PTL remains unclear, the role of prostaglandins has been marked by increasing prostaglandin-endoperoxide synthase 2 (Ptgs2) and reducing 15-hydroxyprostaglandin dehydrogenase (15 Hpgd) expression in the uterus and placenta. The increased Ptgs2 expression may result in the increased production of anti-inflammatory prostaglandins such as PGE 2, PGD 2 and 15d-PGJ 2 as described in other studies [103–105]. Another potential mechanism by which 15-epi-lipoxin 4 may contribute to reducing newborn mortality may be promoting fetal lung maturation. PGE 2 plays a role in regulating the production of fetal pulmonary surfactant. In addition, the administration of the synthetic 15-epi-lipoxin A4 analogue restored surfactant protein C expression in human fetal lung in a bleomycin-induced pulmonary fibrosis model [106], which supports the hypothesis that lipoxin administration may regulate lung surfactant production.

## Conclusion

LXA4, as an arachidonic acid metabolite, is an important component of the inflammatory processes necessary for a proper menstrual cycle, embryo implantation, pregnancy and delivery. Its level in the luteal phase is high, while in the follicular phase it decreases, which coincides with an increase in estradiol concentration with which it competes for the receptor. LXA4 inhibits the progression of endometriosis by participating in the endometrial neuroregulation process. However, during the peri-implantation period, before pregnancy is confirmed clinically, high levels of LXA4 can contribute to early pregnancy loss and may cause miscarriage. After implantation, insufficient LXA4 levels contribute to incorrect maternal vessel remodeling; decreased, shallow trophoblastic invasion; and the immuno-energetic abnormality of the placenta, which negatively affects fetal growth and the maintenance of pregnancy. Defective implantation leads to adverse pregnancy outcomes such as spontaneous miscarriage, fetal growth restriction and pre-eclampsia, which are severe and dangerous perinatal complications. Moreover, the level of LXA4 increases in the final stages of pregnancy, allowing vessel remodeling and placental separation. However, a premature LXA4 level increase before the onset of labor contributes to PTL. Although no human studies have been performed so far, the cell and animal model study results suggest that LXA4 will be used in obstetrics and gynecology soon. On the basis of the presented evidence, supplementing the diet with omega 3 PUFA in high-risk pregnancies should become a priority, and the inclusion of endogenous lipoxin analogues may be a key solution for many pregnancy pathologies.

## Structure of the underlying research

**Fig. 2:**
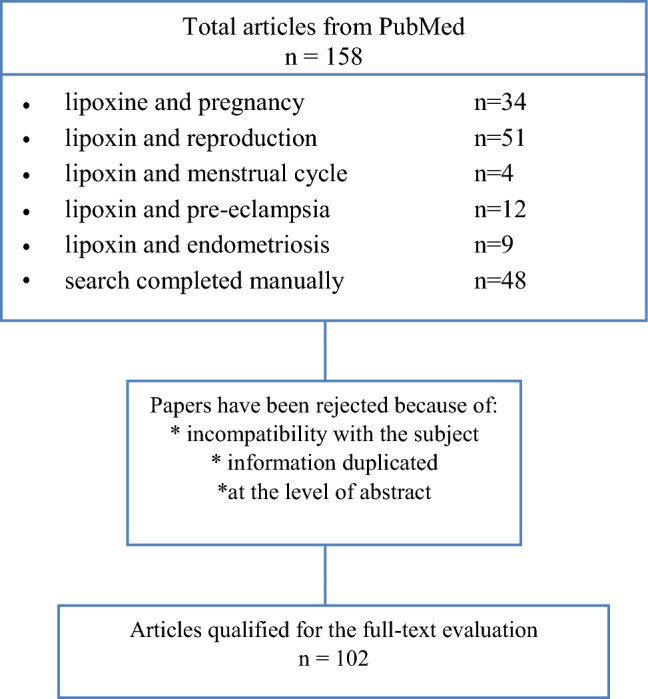
Flow-chart—data collection and study protocol

The present review evaluates the above-mentioned topics considering the literature published up to 31 December 2019. A systematic literature search has been conducted based in the PubMed and Embase database. The passwords were checked on terms: lipoxin and pregnancy. These terms were combined with endometriosis, menstrual cycle, implantation, pre-eclampsia, fetal growth restriction, preterm labor. Studies that were not in English language, letters to editor, abstracts to conferences were excluded as shown on flow chart (Fig. 2). All included studies were screened and discussed by the authors until a general consensus was reached.
